# Depoliticize Human Immunodeficiency Virus Infection: A Commentary

**DOI:** 10.1155/S1064744994000426

**Published:** 1994

**Authors:** Marvin S. Amstey

**Affiliations:** Department of Obstetrics and Gynecology University of Rochester School of Medicine and Dentistry Highland Hospital, 1000 South Avenue Rochester NY 14620 USA

## Abstract

Public-health policy is inconsistent in its approach to the sexually transmitted disease human immunodeficiency virus (HIV). Nearly every health agency has politicized the reporting, finding, and contacting of HIV cases. There is  also no consistency among the various state health departments and the various federal health agencies. Until we have a uniform health policy that treats HIV infection as every other reportable sexually transmitted disease, we will make little progress toward controlling its inevitable increase in both cases and costs.


Infectious Diseases in Obstetrics and Gynecology 2:71–75 (1994)
© 1994 Wiley-Liss, Inc.

### Depoliticize Human Immunodeficiency Virus Infection: A Commentary

**Marvin S. Amstey**

*Department of Obstetrics and Gynecology, University of Rochester, School of Medicine and Dentistry, Highland Hospital, Rochester, NY*

ABSTRACT

**Public-health policy is inconsistent in its approach to the sexually transmitted disease human immunodeficiency virus (HIV). Nearly every health agency has politicized the reporting, finding, and contacting of HIV cases. There is also no consistency among the various state health departments and the various federal health agencies. Until we have a uniform health policy that treats HIV infection as every other reportable sexually transmitted disease, we will make little progress toward controlling its inevitable increase in both cases and costs.** © 1994 Wiley-Liss, Inc.

KEY WORDS
Public health, HIV, AIDS, government policy

Public policy, public-health policy, and government policy all diverge when dealing with human immunodeficiency virus (HIV)-infected individuals and groups. This divergence itself is reason enough for the lack of effective control of this epidemic. This article examines why some of these approaches are counterproductive and, in so doing, makes a case for a return to accepted and demonstrated public-health measures. Even within the public policy-making bodies, there is divergence of opinions and approaches to such important questions as: Should testing be generalized and not just voluntary? Should partners be notified by public-health authorities? Should infected incorrigibles be isolated? Should government policy alter research and funding plans to mount a "war on acquired immunodeficiency syndrome (AIDS)" as it did with the "war on cancer"?

The public perception of AIDS is that it is a major health problem worse than any other disease. The National Leadership Coalition on AIDS found that 50% of Americans list AIDS as their greatest concern; only 7% are concerned about heart disease and 32% are concerned about cancer.[1] The reality is that many more people will die of these 2 disease groups than of AIDS. While AIDS is high on the list of diseases measured in years of life forfeited, so are automobile accidents and murder. Thirty-three percent of Americans will die of heart disease; 24% will die of cancer; and only 1.5% will die of AIDS.[1] For all the terrible perceptions about AIDS and the extraordinary press coverage of this disease, AIDS is only "a small blip" in America's vital statistics.[2] Nonetheless, discussions about AIDS are given high exposure in specific population segments including health-care workers and certain highly skilled, educated, and vocal groups, such as members of the art and fashion industry. The netherworld of drugs and those who live in poverty and within our inner cities also have intelligent and vocal spokespersons.

Despite the very small percentage of people in the general population who die of AIDS, it is now in first place as a killer of American men aged 25–44 years and the fourth leading cause of death among women of the same age.[3] In New York City, AIDS is the leading cause of death among

**Address correspondence/reprint requests to Dr. Marvin S. Amstey, Department of Obstetrics and Gynecology, University of Rochester, School of Medicine and Dentistry, Highland Hospital, 1000 South Avenue, Rochester, NY 14620.**

Received April 20, 1994
Accepted June 15, 1994

women between 20 and 39 years of age. Several studies suggest that, by the year 2000, 50% of the people in this country who are HIV positive will be women.[4] It is well known that the situation worldwide is worse than in the United States, particularly with regard to heterosexual transmission, but this article concentrates on the political responses to this infection in the United States only.

These recent statistics about the rising HIV-infection rate account for the advocates of special-interest groups politicizing a viral disease out of proportion to its place in the hierarchy of vital statistics. Since the onset of the AIDS epidemic in 1981, 204,000 Americans have died of AIDS and its complications, with about 60,000 dying in the current year. In this same year, approximately 1.5 million Americans will die of heart disease and nearly 1 million will die of cancer. Nixon's attempt to politicize cancer and end its devastation by throwing money at the problem and placing the planning and scientific supply-and-demand equation in the hands of politicians failed. The same approaches are taking place with regard to AIDS: the appointment of an AIDS "czar" and the creation of an Office of AIDS Research (OAR) within the National Institutes of Health (NIH) (discussed below) are but 2 examples. These 2 examples are also illustrative of the divergent approaches of government. The "czar" is an administrative appointment; the OAR is a legislative mandate. Who does what? The divergence of funds from traditional research mechanisms or the addition of funds outside the usual scientific channels can only result in the same failure.

Historically, the only political response to an infectious disease was to ignore it or "stamp it out." The best examples of the latter were a state's responses to the plague and syphilis. Authorities quarantined and then burned whole communities harboring plague during the Middle Ages. The history of the repression and ghettoization of prostitutes speaks of political attempts to control syphilis.[5,6] The AIDS epidemic has generated an impressive outpouring of state mandates and prohibitions beyond anything in the past. This has led to the divergence from a public-health approach to finding and controlling a disease to a political approach which is based upon emotion and opinion, not upon scientific and epidemiologic data. In fact, with the AIDS epidemic, the politicians weighed in first by prohibiting the collection of immunologic and sexuality data that are essential to predict the patterns of spread and enable effective control measures to be instituted. These restrictions were followed by various and frequent conferences of public-health officials who established guidelines for collecting data and protecting the individual's health (as opposed to the public's health).[7] Only later did practicing physicians express opinions promoting generalized or involuntary testing and partner notification, 2 historic means of disease identification and control. However, legislation and departments of health mandates forbade these practices even in light of the fact that polls have shown that 90% of non-health-care workers believe that all patients admitted to a hospital should be tested.

Lastly, the lawyers have expressed an opinion about how this epidemic should be handled. The New York Bar Association demanded sweeping legislation giving HIV-infected people new protection, e.g., limiting the admissibility of HIV status in divorce proceedings.[8]

The usual truism is that, when a topic becomes very controversial and the President wishes to avoid making a decision, he appoints a presidential commission. One could argue that the ultimate politicization of a disease is to appoint a presidential commission, which is as far removed from the scientific method as one could wish. In the case of AIDS, not only was the commission a presidential one, but Congress also wished to avoid any decision-making. This led to the National Commission on AIDS which was established by Congress in 1988 under the Health Omnibus Program Extension (HOPE) Act. Like most commissions, this group of 15 people was to study the problem and make recommendations to Congress. In addition, nearly every state has created a gubernatorial commission to study AIDS.

One of the first activities of the National Commission was to travel worldwide to verify that AIDS occurs elsewhere. They concluded that one cannot understand AIDS outside the context of racism, homophobia, poverty, and unemployment.[9] However, no one had wrapped gonorrhea, syphilis, hantavirus, or lyme disease with such baggage before trying to identify cases and seeking some prevention. These infections were dealt with by public-health authorities and the scientific method, not by presidential or congressional commissions.

Goldman and Stryker[9] have asked if HIV is a "special" infection for which informed consent for testing and limitations on disclosure are required. From a public-health point of view, the answer is "no." The Commission believes that the answer is "yes" because HIV-infected people are "shunned and stigmatized." While this is true among large groups within our population, the same could be said about leprosy, which is no longer quarantined, and for syphilis, which is more prevalent and growing faster than AIDS. While HIV infection may be exceptional in morally relevant ways, it is not exceptional in any public-health way. Confidentiality is the common denominator among these and other diseases; stigmatization does not have to occur with proper confidentiality and legislative protection such as that guaranteed by the recently enacted Americans With Disabilities Act. The Commission's error is believing that HIV infection is qualitatively different from other infectious diseases. It is not, and it should not be treated as such.

Cost is another argument presented by the Commission against universal testing for HIV infection. They argue that mass screening programs are prohibitively expensive. While this may be true using current techniques and volumes of tests, the cost/test would shrink with massive testing and simpler tests. More importantly, no thoughts about cost exist for syphilis screening, phenylketonuria (PKU) screening for newborns, or for the anticipated high cost of mass screening for newly identified genetic diseases. If federal or state governments can negotiate for the price of certain vaccines, they could negotiate for the price of HIV screening so that the cost-benefit analysis works.

An extension of the argument about generalized testing is the question of what to do with the information. Dr. Osborne, chairman of the AIDS Commission, believes that an individual's knowledge about his/her HIV status will not change behavior; the behavior must change first.[10] All logic refutes such an argument. While 100% of any population will not change behavior upon knowing if they are HIV positive, certainly some percentage will. Even if 25% altered their behavior, both sexual and drug related, a large fraction of virus would not be transmitted and a large number of uninfected people would be protected.

The most common method of HIV transmission in this country today is needle sharing by intravenous (IV)-drug abusers. Once again, a political response interferes with interrupting this means of HIV transmission. Virtually every state has laws that make it illegal to possess needles and syringes without a prescription. The federal government's response to needle distribution is the usual reflex from the Drug Enforcement Agency (DEA): "No." One of the best examples of the benefits from needle exchanges is the comparison of the HIV-infection rate among prostitutes in 2 cities of equal size and equal economic situations. Newark, NJ (without needle exchanges), has an HIV-infection rate among prostitutes of nearly 50%; Liverpool, England, has a rate of only 1%, but they have an active, mobile needle-exchange service.[11] In other words, a scientific, epidemiologic approach is succeeding, while a political response has resulted in failure.

Another example of a political response to the AIDS epidemic was the passage of the Women's Health Equity Act by Congress in 1991.[12] Some women believed they were excluded systematically from research funding and other health-care funds. The political activism of concerned feminist groups resulted in this act which prompted the Centers for Disease Control (CDC) to add 2 criteria to the definition of AIDS: cervical cancer and repeated candida infections in HIV-infected women, causing the redistribution of some funds. This kind of response has done nothing to control the spread of this infection. Certainly, women are equal partners in this disease, but a political response—a congressional mandate—does not accomplish what a scientific approach toward studying female infections, infected pregnant women, and perinatal transmission of the virus does for the long-term understanding and control of this infection.

Politicization of AIDS has undermined the customary scientifically accepted approaches to clinical research. Vocal activists have short-circuited the randomized, blinded, prospective studies traditionally relied upon by the scientific community. The consequence of this is that drugs are bypassing the usual phases of clinical research. This behavior has led to the mistaken impression that azidothymidine (AZT) has prophylactic benefits, postponing the development of AIDS. Unfortunately, newer drugs are also entering the same short-circuited pipeline, diminishing the ability of scientists to determine their worth. Less stringent criteria for research and subject selection may be politically correct, but they are not scientifically correct.

The third arm of a traditional public-health approach to disease control (along with case finding and partner notification) is education. The public must be made aware of the organism, its mode of transmission, and the current means of preventing that transmission. Even this public-health practice has been compromised by politicians. In 1986, the CDC put restrictions on its grants to private community-based organizations so that they could use money only for educational materials "unoffensive to most educated adults."[13] The U.S. District Court struck down this language as unconstitutionally vague. This may be the ultimate political intervention: a U.S. court countering a U.S. public-health agency's remarks in terms of the Constitution! As one CDC official put it: "We've got to act now, damn it. We have an epidemic that is killing more people than have died in all American wars, including the Civil War, and we're still worried about whether we can talk about condoms or not."[13]

The CDC proposed to educate teenagers and prostitutes and sent their plans to the Department of Education for review. They were rejected by the Reagan Administration, which suggested that all we needed to tell teenagers about AIDS was, "Don't have sex." The only thing needed for prostitute education was to tell them, "Get another job."[14] Not only was a public-health educational plan compromised by politics, it was rendered totally ineffectual. Once again in the AIDS story, the public's health was undermined by politics, a particularly bad brand of politics.

One of the most pernicious effects of the current debate about AIDS is the political control of federal research funds. An activist group, Treatment Action Group (TAG), produced a 200-page critique of AIDS research by NIH. This report received serious attention in the Senate. Proposed legislation would strengthen the OAR by giving it control over NIH's $1.3 billion AIDS budget after 5 years, thereby adding another layer of bureaucracy.[15] Furthermore, it would allow the politicization of AIDS to intrude into basic research in addition to clinical research. It is fair, I believe, to even question the level of federal funding. The AIDS budget is approximately 10% of the entire NIH budget. On a dollar-per-case basis, this figure is very much out of line with where AIDS is in the hierarchy of our national vital statistics.

Ignorance has fueled the promulgation of other equally harmful political measures: legislation requiring HIV-infected physicians to inform their patients; a Florida bill requiring health departments to disclose to school boards the names of children and employees with AIDS; expulsion of HIV-positive students from school in at least 10 different countries; and the denial of work to HIV-positive pilots by a Swedish airline. All of these measures do nothing to prevent transmission of the virus but result from the lack of needed education and the politicization of a disease.

It is evident from the preceding discussion that the public-health approach to AIDS has been overshadowed by a political approach affecting all phases of the infection, from testing to education to research protocols to funding. No other disease has commanded so much political attention, much of it to the detriment of those infected and to those at risk. A misplaced compassion for privacy by a small group of infected individuals and their advocates is increasing the risks to a much larger population.

A return to standard epidemiologic and scientific public-health practices—case finding, contact tracing, and education—offers the best opportunity to control the spread of HIV. As one informed patient said to me recently, "I still don't understand why this is a political disease."

### REFERENCES

1. N.Y. Times, Employee attitudes about AIDS. Nov. 7, 1993, p 25.
2. Greenberg D: The politics of AIDS. Lancet 340:105–106, 1992.
3. N.Y. Times, AIDS is top killer among young men. Oct. 30, 1993.
4. The New Yorker, Women on the edge. April 26, 1993, p 67.
5. Vonderlehr R, Hiller J: The Control of Venereal Disease. New York: Reynal & Hitchcock, 1946.
6. Amstey MS: The political history of syphilis and its application to the AIDS epidemic. Women's Health Issues 4:16–19, 1994.
7. Association of State and Territorial Health Officials: Guide to Public Health Practice: AIDS Confidentiality and Anti-Discrimination Principals, 1988.
8. Associated Press: Democrat and Chronicle, Legal armor for those with AIDS. Rochester, NY, Nov. 7, p. 17A, 1993.

9. Goldman D, Stryker J: The National Committee on AIDS. Kennedy Inst Ethics J 1:339–345, 1991.
10. Osborn J: AIDS: Politics and science. Prev Med 19:744–751, 1990.
11. Suzuki D: Dealing With Drugs. Canadian Broadcasting Co., 1993.
12. Laplatney R: Women and AIDS: The evolution of an epidemic. J NY State Nurses Assoc 22:18–22, 1991.
13. Kosterlitz J: AIDS wars. National Journal, July 25, 1992, p 1727.
14. Westmorland T: The politics and economics of AIDS. J Am Acad Dermatol 22:1303–1305, 1990.
15. Cohen J: Reorganization plan draws fire at NIH. Science 259:753–754, 1993.
